# Mechanism of Prion Propagation: Amyloid Growth Occurs by Monomer Addition

**DOI:** 10.1371/journal.pbio.0020321

**Published:** 2004-09-21

**Authors:** Sean R Collins, Adam Douglass, Ronald D Vale, Jonathan S Weissman

**Affiliations:** **1**Howard Hughes Medical Institute, Department of Cellular and Molecular PharmacologyUniversity of California, San Francisco, CaliforniaUnited States of America

## Abstract

Abundant nonfibrillar oligomeric intermediates are a common feature of amyloid formation, and these oligomers, rather than the final fibers, have been suggested to be the toxic species in some amyloid diseases. Whether such oligomers are critical intermediates for fiber assembly or form in an alternate, potentially separable pathway, however, remains unclear. Here we study the polymerization of the amyloidogenic yeast prion protein Sup35. Rapid polymerization occurs in the absence of observable intermediates, and both targeted kinetic and direct single-molecule fluorescence measurements indicate that fibers grow by monomer addition. A three-step model (nucleation, monomer addition, and fiber fragmentation) accurately accounts for the distinctive kinetic features of amyloid formation, including weak concentration dependence, acceleration by agitation, and sigmoidal shape of the polymerization time course. Thus, amyloid growth can occur by monomer addition in a reaction distinct from and competitive with formation of potentially toxic oligomeric intermediates.

## Abstract 2

Polymerization of the amyloidogenic yeast prion protein Sup35 occurs by monomer addition in a reaction distinct from formation of potentially toxic oligomeric intermediates

## Introduction

Many proteins of diverse sequences, structures, and functions form morphologically similar β-sheet–rich fibrillar aggregates commonly referred to as amyloid (Kelly 1998; Dobson 2001). Amyloid formation is associated with a range of disorders, including neurodegenerative diseases such as Alzheimer's and Parkinson's, and the self-propagating nature of amyloids is thought to underlie prion inheritance. Despite the importance of this process, many questions remain about how amyloid fibers form and grow (Goldberg and Lansbury 2000; Zerovnik 2002; Ross et al. 2003; Thirumalai et al. 2003). While reminiscent of other protein polymerization processes, such as those of actin and tubulin, amyloid formation in most cases does not seem to be well described by simple nucleated polymerization models (DePace et al. 1998; Serio et al. 2000; Padrick and Miranker 2002; Zerovnik 2002; Ross et al. 2003; Thirumalai et al. 2003). Efforts to decipher the underlying mechanism of amyloid conversion have been greatly complicated by the near ubiquitous presence of smaller, oligomeric aggregates during fiber formation and growth (Serio et al. 2000; Bitan et al. 2003; Caughey and Lansbury 2003; Souillac et al. 2003). These oligomers vary widely in morphology, and include spherical, protofibrillar, and annular structures. A growing body of evidence suggests that certain oligomers may be the toxic species that gives rise to amyloid disease (Caughey and Lansbury 2003). Furthermore, it remains an open question whether the fibers themselves are toxic, neutral, or even protective in some cases. However, despite great interest in these oligomers, it is unknown whether they are critical intermediates for amyloid formation or if fibers can form in their absence (Goldberg and Lansbury 2000; Ross et al. 2003; Scheibel et al. 2004).

The yeast prion state [*PSI^+^*], which results from self-propagating aggregation of the translation termination factor Sup35 and leads to a nonsense suppression phenotype, provides an excellent system for studying amyloid fiber formation and prion propagation (Tuite and Cox 2003). Prion inheritance in vivo is mediated by a glutamine/asparagine-rich N-terminal domain (N) and, to a lesser extent, a charged middle domain (M) (Bradley and Liebman 2004). In vitro the NM domain forms self-replicating amyloid fibers (Glover et al. 1997; King et al. 1997), and when introduced into yeast these fibers initiate the [*PSI^+^*] prion state (Sparrer et al. 2000; King and Diaz-Avalos 2004; Tanaka et al. 2004), establishing that the amyloids are in fact the infectious prion element underlying [*PSI^+^*]. De novo NM polymerization is characterized by a long lag phase followed by a cooperative conversion into amyloid. The lag phase can be eliminated by addition of preformed NM fibers. Oligomers similar to those seen during polymerization of other amyloidogenic proteins have been observed during NM fiber formation and even have been seen localized proximal to fiber ends (Serio et al. 2000). The kinetic role of these oligomers, however, remains poorly understood (Scheibel et al. 2004); for example, does amyloid growth occur by capture of oligomeric intermediates at fiber ends, as suggested in earlier studies (Serio et al. 2000)? Here we use a combination of kinetic studies designed to report on specific, well-defined steps in the polymerization reaction together with direct single-molecule fluorescence measurements to explore the mechanism of formation and growth of the infectious NM amyloids.

## Results

**Figure 1:**
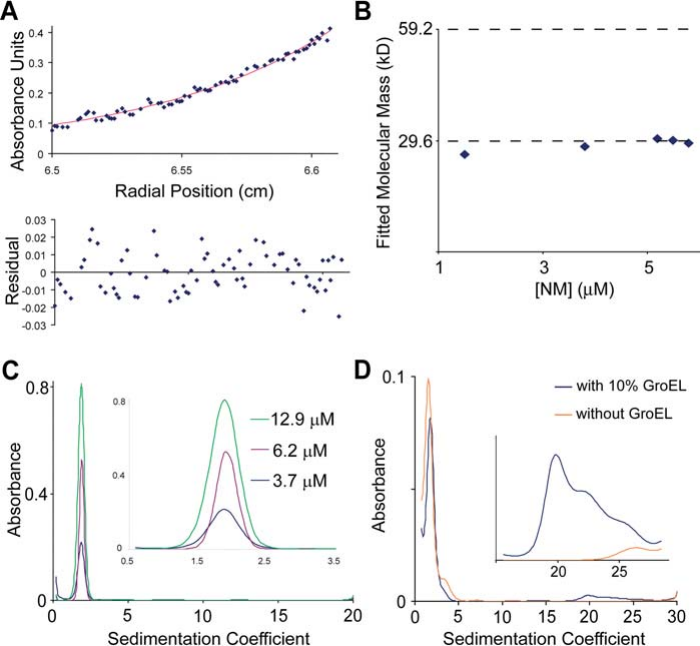
NM Is Predominantly Monomeric (A) Oligomeric state of NM prior to assembly was assessed by equilibrium AUC. Above, raw plot of absorbance versus radial position for 5.8 μM NM at equilibrium, with best-fit line for a single species. Below, residuals from the fit shown above. (B) Equilibrium AUC data from 1.5, 3.8, 5.2, 5.5, and 5.8 μM samples of NM were fit to a single-species model. Shown is fitted molecular mass versus concentration. For reference, dashed lines are shown at the calculated monomer mass (29.6 kDa) and dimer mass (59.2 kDa). (C) Distribution of absorbance versus sedimentation coefficient at the indicated concentrations was obtained from velocity sedimentation data using Sedfit (Schuck et al. 2002). Inset is a magnification of the main peaks. (D) A small amount of a larger complex could be resolved by velocity sedimentation. Distribution of absorbance versus sedimentation coefficient for each of 3 μM NM and 2.7 μM NM plus GroEL (with absorbance equivalent to that of 0.3 μM NM) is shown. Inset is a magnification for sedimentation coefficients between 15.6 S and 28.4 S.

We first examined the distribution of oligomeric species present during NM amyloid formation using analytical ultracentrifugation (AUC). Equilibrium AUC indicated the material was predominantly monomeric, giving fitted average molecular masses of 28.5 ± 1.7 kDa (calculated monomer mass is 29.6 kDa), with no appreciable concentration dependence from 1.5 to 5.8 μM (Figure 1A and 1B). By velocity AUC, which is better suited to resolve a small population of a larger oligomer (Schuck et al. 2002), the vast majority of the material fit well to a single peak in a sedimentation coefficient distribution obtained by direct boundary modeling [c(s)] (Schuck et al. 2002) with a sedimentation coefficient of 1.9 S (Figure 1C). The lack of other detectable peaks is significant, as we were readily able to detect the presence of small amounts of a larger protein complex (10% GroEL) added to an NM sample (Figure 1D). Thus, on the time scale of the 5–20 h needed for the AUC analysis, which exceeds the polymerization reaction times in our present studies, the large majority of NM is monomeric. This has allowed us to examine fiber formation in the absence of significant off-pathway aggregation, which was seen to obscure the underlying kinetics of other amyloid systems (Rhoades et al. 2000; Padrick and Miranker 2002; Souillac et al. 2003). However, our AUC data did not address whether a rare oligomeric species serves as a critical on-pathway intermediate for fiber growth.

**Figure 2:**
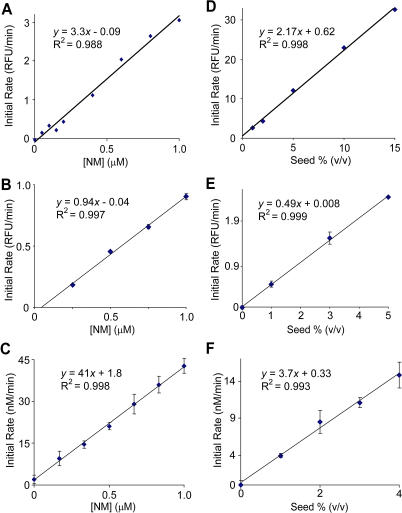
Kinetics of NM Fiber Growth Support a Monomer Addition Model (A) Initial rate of polymerization versus concentration of NM. Soluble NM at the indicated final concentrations was mixed with sonicated fibers (2.5 μM fibers at 5% of final volume) and polymerization was followed by a continuous thioflavin T assay. Rates shown were determined by the initial slopes of polymerization curves. (B) Initial rate of polymerization versus concentration of soluble NM in the presence of sonicated seed (1% of final volume) measured by a discrete thioflavin T binding assay. Error bars throughout represent the standard deviation of at least three measurements. (C) Polymerization of NM labeled with Alexa-647 at a C-terminal cysteine was monitored by the quenching of Alexa-647 fluorescence. The indicated concentrations of soluble NM were mixed with sonicated fibers (4% of final volume), and the initial rate of polymerization was measured. (D) Initial rate of polymerization versus concentration of seed. Soluble NM (2.5 μM final concentration) was mixed with the indicated quantity of sonicated seed. Highly fragmented fibers were used to maximize the absolute rate. Rates were measured as in (A). (E) Initial rate of polymerization versus concentration of seed as measured by discrete thioflavin T binding assay. Initial soluble NM concentration was 2.5 μM. (F) Initial rate of polymerization versus concentration of seed as measured by Alexa-647 fluorescence quenching. Initial soluble NM concentration was 200 nM.

To explore this possibility, we looked at the concentration dependence of the initial rate of growth of soluble NM onto the ends of a well-defined amount of preformed fibers. If a rare oligomer is critical for fiber growth, then mass action dictates that the quantity and rate of formation of such oligomers will be highly concentration dependent. As a consequence, the initial rate of polymerization should depend strongly on the concentration of soluble NM. Using a thioflavin T binding assay that allows continuous measurement of amyloid formation, we measured the rate of amyloid growth after mixing a quantity of soluble NM with a known amount of preformed nuclei. We found that the initial rate of fiber growth was directly proportional to the concentration of soluble NM over a range of 0–1 μM (Figure 2A). This linear dependence of fiber growth on NM concentration was not dependent on the method used to monitor growth (Figure 2B and 2C). We also looked at the dependence of the polymerization rate on seed concentration. As expected, this rate was directly proportional to concentration of fiber ends (Figure 2D–2F), and importantly, the rate was still linear up to a concentration of seed at which half of the soluble material was polymerized in less than 3 min. Therefore, NM in solution is in rapid equilibrium with conformations competent to add to fibers.

**Figure 3:**
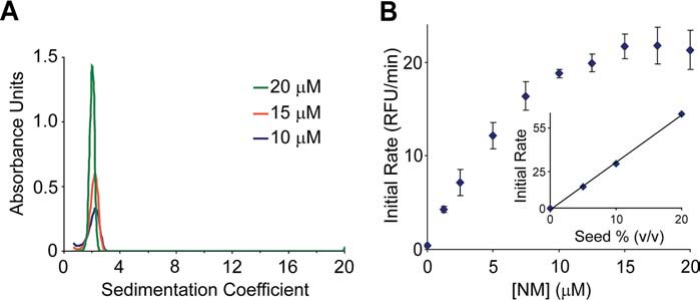
Evidence that a Conformational Conversion following NM Monomer Binding to Fiber Ends Becomes Rate Limiting at High [NM] (A) NM is predominantly monomeric at concentrations up to 20 μM in the presence of a moderate level of denaturant (100 mM urea and 100 mM GuHCl). Oligomeric state was assessed by velocity AUC as in Figure 1C for NM at 10, 15, and 20 μM. (B) Soluble NM was mixed with sonicated fibers in the presence of 100 mM urea and 100 mM GuHCl to minimize off-pathway aggregation at higher [NM], and the initial rate of polymerization was measured as in Figure 2A. Inset, rate of polymerization of 20 μM soluble NM versus seed percentage (v/v). The continued linear dependence of rate on fiber ends indicates that at high [NM], fiber ends remain limiting.

**Figure 5:**
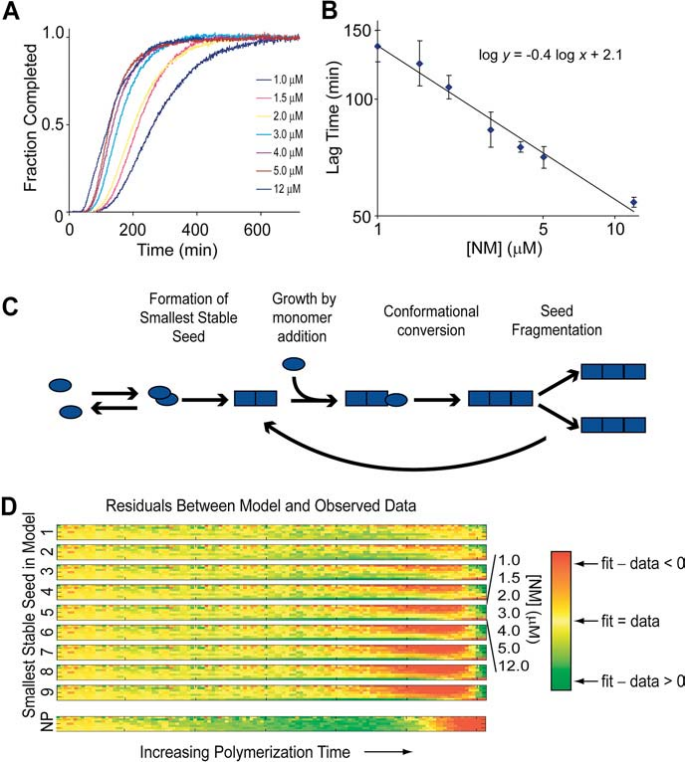
Effect of Fiber Fragmentation on Polymerization Kinetics (A) De novo NM polymerization (1.0–12 μM) followed by a continuous thioflavin T binding assay. (B) Lag time (measured as time to 5% completion of polymerization) versus NM concentration for the polymerizations shown in (A). (C) Schematic of nucleated polymerization with fragmentation. At low concentrations fiber growth is limited by monomer binding, whereas at high concentrations conformational conversion after binding becomes limiting. (D) The de novo polymerization data shown in (A) were fit with a linearized model (Ferrone 1999) that includes nucleation, fiber growth by monomer addition, and fragmentation, assuming the indicated sizes for the smallest stable amyloid species (nucleus size). Plotted are the residuals (best-fit value minus observed data). Each block (labeled at the left by the nucleus size used) represents the residuals from simultaneously fitting all of the data. Each line within the block displays the individual residuals from a single concentration (1 μM [top] to 12 μM [bottom]). Residuals are color coded according to the color key at the right, with red and green indicating large errors and yellow indicating a good fit. Time varies from time zero (farthest left) to the time of 3% completion of polymerization. Below (NP) are residuals from fitting the same data using a simple nucleated polymerization model. Note the systematic deviations from the model for large nucleus sizes and for NP.

Interestingly, at higher NM concentrations (>10 μM), the rate of fiber elongation shows a weaker-than-linear dependence on NM concentration. Accumulation of off-pathway aggregates, which we can observe at high [NM], could contribute to such an effect. However, in the presence of moderate levels of denaturant (100 mM guanidine hydrochloride [GuHCl] and 100 mM urea), NM remained monomeric up to at least 20 μM (Figure 3A), and under these conditions we still observed that the rate of polymerization becomes largely independent of the concentration of NM (Figure 3B). This suggests that a conformational rearrangement of NM after binding to fiber ends becomes rate limiting at high NM concentrations (see Figure 5C). As would be expected if unconverted NM is occupying fiber ends, even at the highest NM levels the rate of polymerization remains linearly dependent on the amount of seed added (Figure 3B, inset). This conformational rearrangement may be analogous to the locking step observed in Aβ polymerization (Esler et al. 2000; Cannon et al. 2004), although here it occurs on a faster time scale. Estimates from growth rates measured by atomic force microscopy (AFM) indicate that the rearrangement occurs within ∼1 s (see Materials and Methods), a time similar to that seen for the folding of comparably sized β-sheet–rich proteins (Reid et al. 1998).

**Figure 4:**
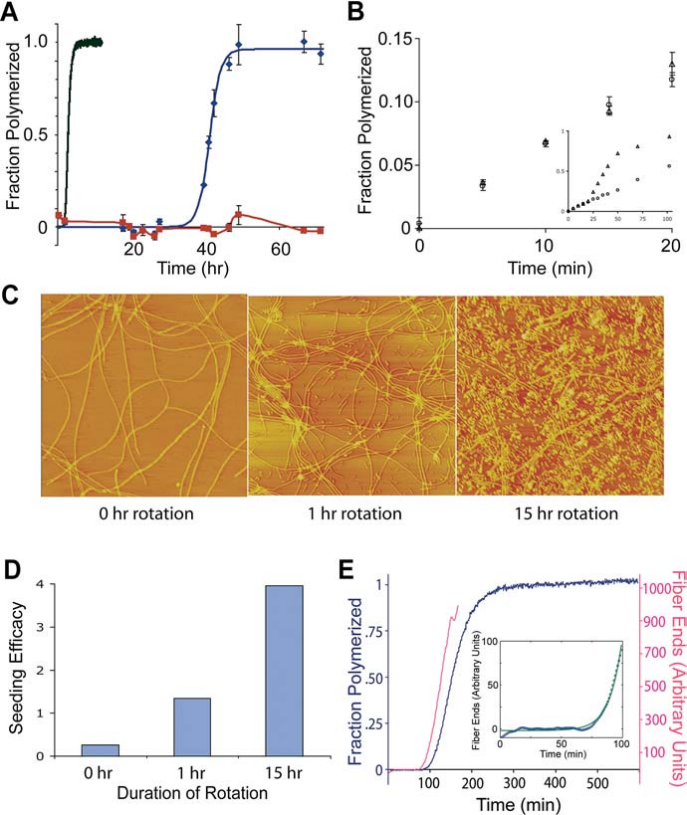
Agitation Causes Fiber Fragmentation (A) Dependence of de novo polymerization reactions on degree of agitation. Polymerization in a microplate with shaking every minute (line only) was followed by thioflavin T fluorescence. Polymerization in a test tube disturbed only by pipetting to take samples for measurement (diamonds) was measured by Congo Red binding. Polymerization in a microplate with absolutely no agitation (multiple samples were started in parallel and no sample was measured more than once) (squares) was measured by Congo Red binding. (B) Effect of agitation on elongation rate. Identical reactions with 5 μM soluble NM and 2% (v/v) seeds were grown with (triangles) or without (circles) agitation. The seeds were made fresh and sheared by passing through a 25-gauge needle ten times. Polymerization was assayed by discrete thioflavin T measurements. Inset, identical reactions followed for 100 min. (C) Effect of agitation on fiber lengths. Long fibers were grown from sonicated seeds in the absence of agitation and then subjected to agitation (end-over-end rotation in a 2-ml tube). AFM images were taken after 0, 1, and 15 h of rotation. Each image is 5 μm by 5 μm. (D) Effect of agitation on seeding efficacy. Fibers from the samples imaged in (C) were used to seed polymerization of 10 μM soluble NM, and the initial rate of polymerization was measured as in Figure 2A. (E) De novo NM polymerization was measured by continuous thioflavin T fluorescence, and relative fiber number was computed as a function of time (see Materials and Methods). Inset, fiber number versus time was fit to an exponential curve for time = 0 to time = 100 min.

A model in which growth of NM fibers is largely limited by the encounter rate of monomer and fiber end still has two puzzling features of the polymerization process to explain. First, like many other amyloid formation reactions, NM polymerization is dramatically accelerated by agitation (DePace et al. 1998; Serio et al. 2000) (Figure 4A), which has been hypothesized to increase the fiber growth rate by breaking up off-pathway aggregates or speeding up diffusion of large on-pathway oligomers (Serio et al. 2000). Neither of these explanations applies to a predominantly monomeric solution where fibers grow by monomer addition. Previous attempts to understand agitation were complicated by the multistep nature of the polymerization process, which includes nucleation, growth, and other steps. We specifically looked at the effect of agitation on the fiber growth step using two identical seeded reactions, one agitated and one undisturbed. Remarkably, we found that seeded polymerizations initially proceeded at exactly the same rate with or without agitation (Figure 4B). However, after extensive growth of the added seeds (∼20 min) the rate of the agitated reaction began to accelerate markedly (Figure 4B, inset), whereas unagitated reactions slow down as the pool of monomeric NM becomes depleted.

At a given NM concentration the rate of polymerization is proportional to seed concentration, and therefore the acceleration seen in the agitated reaction implies that the number of fiber ends is increasing with time, perhaps due to the ability of agitation to fragment long fibers. To test this possibility, we prepared long fibers by allowing prolonged growth of NM onto preformed seeds in the absence of agitation, and then subjected the fibers to end-over-end rotation. Prior to rotation, the fibers were very long and had a weak ability to seed polymerization of monomeric NM. After 1 h of rotation, we saw many more fibers of much shorter length, and the seeding activity had increased 10-fold. Further rotation (14 h) produced still shorter fibers and higher seeding activity (Figure 4C and 4D). Interestingly, a reanalysis of the kinetics of de novo NM polymerization, based on the fact that the rate of polymerization is linearly dependent on the amount of seed present and the amount of monomer remaining (see Figure 2), indicates that the number of seeds increases exponentially during NM polymerization (Figure 4E). Thus, while agitation does not affect the rate of NM addition to fiber ends, it accelerates polymerization by increasing the number of ends through amyloid fragmentation.

The second unusual feature of NM polymerization that must be reconciled with a model of monomer addition is the weak dependence (less than first order) of the length of the lag time on the concentration of NM in unseeded polymerizations (DePace et al. 1998; Serio et al. 2000). This finding, together with the sigmoidal curve shape involving a pronounced lag phase followed by an abrupt increase in the rate of polymerization, provided key evidence against a simple nucleation-polymerization model (DePace et al. 1998; Serio et al. 2000; Padrick and Miranker 2002), which is characterized by an initially parabolic (t^2^) time course (Ferrone 1999). We similarly observed a lag phase that depends on approximately the 0.4 power of initial concentration (Figure 5A and 5B). In other systems, this weak concentration dependence has been attributed to an accumulation of large off-pathway species whose formation is competitive with on-pathway processes (Rhoades et al. 2000; Serio et al. 2000; Padrick and Miranker 2002; Souillac et al. 2003). However, in our experiments NM is predominantly monomeric, so the observed weak concentration dependence is not a simple consequence of the accumulation of off-pathway aggregates.

Previous work from Ferrone (1999) using a linearized model demonstrated that addition of fragmentation to a nucleation-polymerization process could lead to sigmoidal polymerization curves. We explored whether fragmentation could also explain the weak concentration dependence using both numerical integration and direct fitting of the data to a linearized model. We modeled a simple polymerization process involving nucleation, growth, and fragmentation (Figure 5C). Numerical integration confirmed the expected strongly sigmoidal curve shape, but we also found that fragmentation greatly reduced the concentration dependence. For example, a nucleus size (the number of monomers in the smallest stable amyloid species) of six gives a third-order concentration dependence in nucleated polymerization but only approximately a first-order dependence with fragmentation, and the apparent concentration dependence decreases further for smaller nucleus sizes. We additionally tried fitting data to the linearized analytical solution which is valid for early parts of the polymerization (Ferrone 1999; we restrict ourselves to the first 3%) and only requires fitting two parameters. Fixing nucleus size and fitting polymerization curves at seven concentrations, we obtained residuals of fits at a series of potential nucleus sizes (Figure 5D). Although more direct measurements are needed to define the exact size of the minimal stable seed, both approaches suggest a small nucleus size (three monomers or smaller), consistent with recent observations on polyglutamine polymerization (Chen et al. 2002).

**Figure 6:**
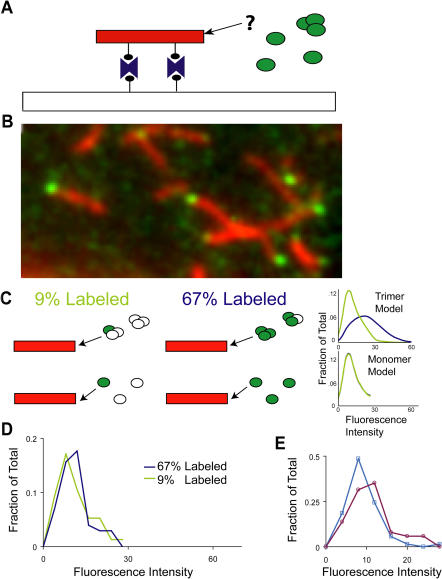
Fundamental Unit of Addition for NM Fiber Growth Determined by TIRF (A) Schematic of experimental setup. Cy5-labeled fibers (red) were attached to the microscope slide via biotin-streptavidin linkage. Cy3-labeled NM (green) was added in solution. (B) TIRF image of fibers (red) grown by addition of 200 nM NM (67% labeled with Cy3 [green]) for approximately 1 h. Cy3 fluorescence at fiber ends in this image represents multiple addition events. (C) Schematic of expected results if oligomers (top, trimer model shown for example) or monomers (bottom) are added to fiber ends. Graphs at the right show simulated data of fraction of additions versus fluorescence intensity of addition. Note that for the monomer model, the simulated intensities are the same whether 9% or 67% of the soluble NM is labeled. (D) Observed data: fraction of observed events versus fluorescence intensity of events. Intensities of Cy3 spots appearing at fiber ends were measured. (E) Intensities of Cy3 spots appearing at fiber ends (circles) and intensities of Cy3 spots that bleached in a single step (squares).

The advent of single-molecule fluorescence technology enabled an independent and more direct way to test whether fibers grow by monomer addition. Previous work (Inoue et al. 2001) established that fluorescent NM fibers attached to a microscope slide could be grown and visualized using epifluorescence. Here we examined fiber growth using total internal reflection fluorescence microscopy (TIRF), which allows single-molecule detection. We attached Cy5-labeled NM fibers to a slide through a biotin-streptavidin linkage and added a solution of Cy3-labeled soluble NM (Figure 6A). Working at a label concentration of 133 nM (200 nM or more total soluble NM) to minimize background fluorescence, we could readily detect addition of individual Cy3 fluorophores at the ends of Cy5-labeled fibers. Two observations indicate that we were monitoring growth events mediated by fiber ends. First, after extended time (∼1 h) at our working concentration, bright Cy3 fluorescence (consisting of many labeled NM molecules) accumulated specifically at fiber ends (Figure 6B). Second, Cy3 addition events at fiber ends were long lived (55% of spots at fiber ends remained visible in the second frame measured 15 s later, whereas in the absence of fiber ends fewer than 20% of spots remained).

By following the intensities of single fluorescent addition events, we could directly determine the size of the unit of addition. Oligomer addition (unlike monomer addition) predicts that the fluorescence intensity of a single addition event should depend strongly on the fraction of NM that is fluorescently labeled (Figure 6C). We prepared NM at both 67% (133 nM label, 200 nM total soluble NM) and 9% (89 nM label, 966 nM total soluble NM) labeling efficiency. Intensities of Cy3 spots that appeared at fiber ends were then measured, and for each degree of labeling, a histogram of intensities was created. Strikingly, we observed that the distribution of intensities was independent of the degree of labeling (Figure 6D). Furthermore, the intensity distribution of the fiber addition events was comparable to that of NM-Cy3, which was confirmed to be monomeric by single-step photobleaching experiments (Figure 6E). Together these data establish that amyloid growth is occurring by the addition of NM monomers onto fiber ends.

## Discussion

Amyloid formation is a ubiquitous feature of polypeptides. However, compared to protein folding reactions, for which a wide range of biophysical, structural, and analytical approaches have provided detailed information on the pathways by which native states are obtained, very little is known about the underlying steps by which amyloid fibers assemble and grow. In large part this is due to the complexity of the amyloid formation reaction, which can involve a wide spectrum of on- and off-pathway intermediates. In the present study, we have used a combination of kinetic analysis designed to look at specific mechanistic steps and single-molecule fluorescence to determine how amyloid fibers of Sup35, the prion determinant of the yeast state [*PSI^+^*], form and grow.

A key finding is that Sup35 NM amyloids grow efficiently by the addition of monomers to fiber ends. We also establish that monomer addition, in combination with fiber fragmentation, accurately predicts the otherwise puzzling features of de novo polymerization kinetics. While it is still possible that oligomers could add to the ends of fibers if allowed to accumulate, we find that monomer addition is rapid and efficient. This monomer addition mechanism can account for the generation of the [*PSI^+^*] translation read-through phenotype in vivo. While division of Sup35 prion particles appears to depend on cellular chaperones including Hsp104 (Ness et al. 2002; Osherovich et al. 2004), both genetic and biochemical evidence suggests their growth is an Hsp104-independent process (Ness et al. 2002; Shorter and Lindquist 2004). Additionally, with approximately 200 seeds in a cell (Cox et al. 2003) and the second-order rate constant we observe (approximately 2 × 10^5^ M^−1^ s^−1^), soluble Sup35 would have a half-life of about 3 min, which is comparable to the time scale of Sup35 translation and much faster than the doubling time of yeast (90 min). Thus, the monomer growth mechanism would lead to the depletion of Sup35 characteristic of the [*PSI^+^*] state (see Materials and Methods). Moreover, the properties of Sup35 polymerization we observe seem particularly well suited to explain the prion phenotype in yeast. De novo nucleation of new fibers is extremely slow, but existing fibers grow rapidly and their fragility may allow them to be divided easily. These features are appropriate for a protein-based switch that is bistable ([*PSI^+^*] and [*psi* ^−^] are both stable states) and whose aggregated state must be amplified exponentially to keep pace with cell division.

Many kinetic features seen for NM polymerization are shared by other amyloidogenic proteins (Uversky et al. 2001; Chen et al. 2002; Padrick and Miranker 2002), suggesting that monomer addition may represent a mechanism of amyloid growth common to other fibers. Whether other amyloids do in fact grow by monomer addition and how the role of oligomeric species in the polymerization process correlates with toxicity remain important open questions. Many of the analytical approaches and experimental techniques used in this work should be directly applicable for exploring these issues in other systems. A broader understanding of the role of oligomeric species in the formation and growth of amyloids will be critical for determining the physiological effects of amyloidogenesis as well as guiding efforts to counteract amyloid toxicity. For example, the conclusion that amyloid growth and oligomer formation can occur in distinct, competitive reactions may help explain the poor correlation between formation of visible aggregates and toxicity in neurodegenerative diseases of protein misfolding (Saudou et al. 1998; Cummings et al. 1999; Goldberg and Lansbury 2000; Wittmann et al. 2001). More speculatively, the finding that fiber growth does not require oligomeric intermediates raises the possibility that agents designed to promote direct fiber formation, by disfavoring oligomer formation, may help prevent the accumulation of potentially toxic oligomeric intermediates.

## Materials and Methods

### 

#### Reagents

Cy3 mono maleimide Gold and Cy5 mono maleimide Gold were purchased from Amersham (Little Chalfont, United Kingdom). Alexa Fluor 647 C_2_ maleimide was purchased from Molecular Probes (Eugene, Oregon, United States). Streptavidin was purchased from Molecular Probes. Biotinylated BSA and thioflavin T were purchased from Sigma (St. Louis, Missouri, United States). Biotin-PEAC_5_-maleimide (6-{N′-[2-(N-Maleimido)ethyl]-N-piperazinylamido}hexyl D-biotinamide, hydrochloride) was purchased from Dojindo (Gaithersburg, Maryland, United States). Alkali-soluble casein (5%) was purchased from Novagen (Madison, Wisconsin, United States).

#### Protein expression

Sup35 residues 1 to 254 (NM) C-terminally tagged with 7×-histidine were purified as reported previously (DePace et al. 1998; Tanaka et al. 2004).

#### Fluorescence labeling

NM with a single cysteine inserted after the polyhistidine tag was labeled by Cy3 mono maleimide Gold, Cy5 mono maleimide Gold, or Alexa Fluor 647 C_2_ maleimide (10 equivalents) in 25 mM sodium phosphate buffer containing 6 M GuHCl and 0.1 mM TCEP (pH 8.0) at 4 °C overnight with 67% efficiency. Efficiency of labeling was determined from absorbance at 275 nm and at the excitation maximum of the dyes, correcting for absorbance of the dye at 275 nm. Biotinylated NM was made analogously using biotin-PEAC_5_-maleimide (2.5 equivalents) with 1 mM TCEP, obtaining about 20% efficiency.

#### AUC

Both velocity and equilibrium AUC were performed in a Beckman Optima XL-A analytical ultracentrifuge using an An60Ti-Rotor at 20 °C. Protein was in buffer C (5 mM potassium phosphate and 150 mM sodium chloride [pH 7.4]). Velocity sedimentation was analyzed at a speed of 40,000 rpm at NM concentrations of 3.7, 6.2, and 12.9 μM (determined by absorbance at 275 nm). Data were collected with three replicates at radial steps of 0.003 cm and scans every 9 min. Data were analyzed with Sedfit using the c(s) method (Schuck et al. 2002). Equilibrium sedimentation was analyzed at a speed of 23,300 rpm for concentrations of 1.5, 3.8, 5.0, 5.5, and 5.8 μM. Data were analyzed with Sedphat (Schuck 2003) by fitting each curve individually to a single species model. Sedfit and Sedphat are available at http://www.analyticalultracentrifugation.com.

#### Polymerizations

For continuous thioflavin T assay measurements (see Figures 2A, 2D, 3B, 4A, 4D, 4E, 5A, 5B, and 5D), concentrated stocks of NM stored in either 6 M GuHCl (fluorescently labeled NM and NM used for Figures 2C, 2F, and 6) or 4 M GuHCl and 4 M urea (NM used for all other experiments) were diluted at least 200-fold into buffer C. In order to compare polymerizations done at different concentrations of NM, residual denaturant concentrations were equalized for all samples in each experiment. Reactions consisted of 100 μl of protein in buffer C plus 100 μl of 25 μM thioflavin T in 50 mM glycine (pH 8.5). Seed was added immediately before observation. Fluorescence was monitored in a 96-well fluorescence plate reader (Molecular Devices, Sunnyvale, California, United States; 442 nm excitation and 483 nm emission). Reactions were carried out at 25 °C either without (for seeded reactions) or with (for unseeded reactions) 3 s of shaking between each measurement. Unseeded polymerizations were done in the presence of a small amount of soluble casein (0.02%), as empirically that condition gave flatter plateaus at the end of the reaction.

Discrete thioflavin T assay measurements (see Figures 2B, 2E, and 4B) were performed as continuous measurements, except that polymerization proceeded in buffer C in the absence of thioflavin T. At indicated time points, a 100-μl aliquot of polymerization reaction was mixed with 100 μl of thioflavin T for measurement. Reactions were either undisturbed or rotated end-over-end in a 2-ml tube as indicated.

For polymerizations followed by Alexa Fluor 647 fluorescence (see Figures 2C and 2F), the fluorescence of a 200-μl volume containing the indicated concentration of labeled NM and the indicated quantity of sonicated fibers was measured over time in a 96-well fluorescence plate reader (Molecular Devices; 650 nm excitation and 670 nm emission). Fluorescence was partially quenched (approximately 56% of fluorescence lost) upon polymerization. Rates of polymerization were determined by taking the initial slope of the polymerization curve divided by the total change in fluorescence multiplied by the concentration of the sample. Wells in the microplate were blocked with 5% casein and washed with water prior to polymerization experiments to minimize adsorption of NM to the sides of the wells.

Seeds used were produced by polymerization of 2.5 μM NM followed by sonication with a Fisher Scientific Sonic Dismembrator (Model 500) fitted with a microtip for 60 s (for all experiments except that shown in Figure 4B) or sheared by passing through a 25-gauge needle ten times (for Figure 4B). Sheared rather than sonicated fibers were used for examining the effect of agitation on fiber growth because longer fibers were seen to break more easily than shorter fibers.

#### AFM

AFM samples were prepared and analyzed essentially as previously described (DePace and Weissman 2002). All images were taken using tapping mode on a Digital Instruments Multimode AFM, Nanoscope IIIa controller, and Micromasch NSC15 tips. For fiber images (see Figure 4C), 20 μl of fibers (either 2.5 or 5.0 μM total NM) was deposited on mica disk for 20 s. The disk was then washed twice with 160 μl of distilled deionized water and aspirated until dry.

For estimating fiber growth rates (see below), new growth of unlabeled NM off of preformed fibers made from NM tagged with an HA epitope was measured as previously described (DePace and Weissman 2002). The HA epitope allows the initial seeds, but not new growth, to be labeled with antibody after the fibers are deposited on the mica.

#### Computer simulation, curve fitting, and computation

Numerical integration was performed using Euler's method implemented in a C program (code available upon request). The following is a general description of the strategy used for numerical modeling. A set of differential equations was written to account for the following steps: formation of a new stable nucleus from a defined number (n) of monomers, growth of fibers by monomer addition (limited by the rate of encounter of monomer and fiber end), and production of new fibers by fragmentation of existing fibers. Fiber growth was modeled to be irreversible (i.e., fibers do not depolymerize) (DePace and Weissman 2002), because experimentally the critical concentration for growth appeared to be very small (less than 50 nM), if one exists. Fragmentation was modeled as a length-dependent process (long fibers break more easily than short fibers), and to simplify calculations, it was assumed that no fiber breaks to yield a fragment as small or smaller than the size of the smallest stable nucleus. This is likely to be true for the large majority of fragmentation events because of the tendency of fibers to break closer to their center rather than their ends and because of the low propensity of short fibers to break (Hill 1983). Concentrations of monomers and fibers of each length from n to 3,016 monomers were modeled as a function of time. An upper bound for fiber length was necessary to make the calculations possible, and the total of 3,016 was chosen because increasing this bound had no measurable impact on results.

Variables and parameters used for modeling were the following: *t* = time; *x* = concentration of monomers; *y*_i_ = concentration of fibers containing i monomers; *y* = concentration of all fibers of length n or greater; n = the number of monomers in the smallest stable species; BreakDep = the power to which the rate of fragmentation of a fiber depends on its length. For all simulations this value was set to 3, based on theoretical estimates for polymers (Hill 1983), although the results were robust to changes in this parameter. The BreakDep parameter was included to model the length dependence of fragmentation:



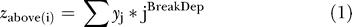



Here, j represents fiber length (the number of monomers in a fiber) and the sum is taken over all values of j greater than or equal to (i + n + 1). The *z*_above(i)_ term accounts for fragmentation of all fibers long enough to break and give a resulting fiber of length i. The sum begins at j = (i + n + 1) because no smaller fiber could break to give a fragment of size i, leaving another fragment of at least size (n + 1).

Differential equations for modeling contain the following terms: k_nuc_ = effective fiber nucleation rate constant; k_growth_ = fiber elongation rate constant; k_break_ = fiber fragmentation rate constant. The equations are as follows.

For monomer concentration:



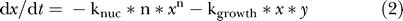



The k_nuc_*n*x ^n^ term accounts for the loss of n monomers during the formation of each stable nucleus. Stable nuclei are modeled to form at a rate of k_nuc_*x ^n^. The k_growth_*x*y term accounts for the loss of one monomer for each fiber elongation event; each fiber grows at a rate of k_growth_*x and there are *y* total fibers.

For the smallest stable species:



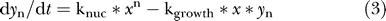



The k_nuc_*x ^n^ term accounts for the spontaneous formation of new nuclei. The k_growth_*x*y_n_ term accounts for the disappearance of fibers of length n as they grow into longer fibers.

For short fibers (of length less than or equal to twice the size of the nucleus plus one monomer):



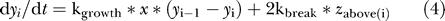



Fibers of length i are created by elongation of shorter fibers at a rate of k_growth_*x*y_i−1_ and lost by growth into longer fibers at a rate of k_growth_*x*y_i_, accounting for the first term. The second term accounts for formation of fibers of length i by fragmentation of longer fibers (see description of *z*_above(i)_ above). There is no loss of fibers of these lengths from fragmentation because they are assumed to be too short to break.

For fibers longer than twice the size of the nucleus plus one monomer:



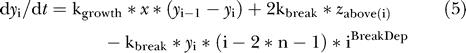



The first two terms are the same as for the short fibers. The additional term accounts for fragmentation of fibers of length i into shorter fibers. This term is arrived at in the following way: k_break_ is a rate constant for the fragmentation process, *y*_i_ is the number of fibers of this length, (i − 2*n − 1) is the number of points along the length of the fiber where it could break, and i^BreakDep^ accounts for the tendency of longer fibers to break more easily than shorter fibers (see the description of BreakDep above). There are (i − 2*n − 1) places the fiber could break because there are (i − 1) monomer-monomer interfaces in a fiber of length i and breaking at any of the n interfaces closest to either end of the fiber would give one fiber of size n or smaller.

Curve fitting of experimental data to the linearized model of Ferrone (1999) was performed using a Levenberg-Marquardt least squares fitting method implemented in MATLAB (The Mathworks, Natick, Massachusetts, United States). Data was fit to the equation *y* = A(cosh (B*t*) − 1), where *t* is time and A and B are parameters to be fit with the restriction that AB^2^ scales as [NM]^n+1^ (where n is the number of monomers in the smallest stable species).

Relative fiber number (see Figure 4E) was computed in the following way: If *z* = total amount of NM in amyloid, and z_final_ = total amount of NM in amyloid at the end of the reaction, then relative fiber number = (d*z*/d*t*)/(z_final_ − *z*). Values for *z* were measured by thioflavin T fluorescence (continuous thioflavin T assay). The equation used for relative fiber number comes from the observation (see Figure 2) that the bulk growth rate (d*z*/d*t*) is proportional to both fiber concentration (relative fiber number) and soluble NM concentration (z_final_ − *z*). The relative fiber number versus time was fit to an exponential (*y* = Ae^b*t*^) by nonlinear least squares regression as described above (see Figure 4E).

#### TIRF

Single-molecule imaging was performed using objective-type TIRF illumination configured on a Zeiss Axiovert 200M (Carl Zeiss, Inc., Zurich, Switzerland), and controlled by the QED in vivo software package (Media Cybernetics, Silver Spring, Maryland, United States). Images were acquired digitally with a Mega-10 intensified CCD camera (Stanford Photonics, Stanford, California, United States) and analyzed using ImageJ (National Institutes of Health, Bethesda, Maryland, United States) and MATLAB software. Samples were analyzed in glass-bottom microwell dishes (MatTek, Ashland, Massachusetts, United States; catalog #P35G-1.5-14-C) which were prepared by application of 60 μl of biotinylated BSA (1 mg/ml) for 20 min followed by washing with buffer, application of 60 μl of streptavidin (0.2 mg/ml) for 20 min, washing, application of 100 μl of casein (5%) for 30 min to 2 h, washing, application of 40 μl of Cy5-labeled fibers for 10 min, and finally washing with buffer. The fibers were prepared in a 300-μl reaction with 1% (v/v) seed (sonicated from a 2.5 μM NM reaction). The sonicated seed was grown for 15 min with NM at 2.5 μM (5% biotinylated NM, 20% Cy5-labeled, and 75% unlabeled). The seed was diluted 2-fold and sheared with a 200-μl pipet tip before application to the slides. The slides were kept in buffer until use. Buffer was removed and Cy3-labeled NM (200 nM, 67% labeled; or 955 nM, 9% labeled) was added to the slide immediately before viewing. For addition studies, a Cy5 image was taken to locate the fiber ends and then images were taken every 15 s in the Cy3 channel to look for events at fiber ends.

#### Estimate of maximum fiber growth rate

AFM analysis established that NM fibers grow at an average rate of 100 nm/min at a soluble NM concentration of 2.5 μM and can grow at least a factor of two faster at higher concentrations. Estimating one monomer per 3 nm of length in a fiber (from the approximate monomer volume and a fiber diameter of 4.5 nm from AFM) gives a rate of at least one per second. This is a conservative estimate (the real rate may be faster) because larger fiber diameters have been measured by electron microscopy (Kishimoto et al. 2004), indicating that fibers may have more than one monomer per 3 nm of length.

#### Estimating a half-life for Sup35 in a cell

We observe a second-order rate constant for fiber elongation of approximately 2 × 10^5^ M^−1^ s^−1^, and estimating 200 seeds (Cox et al. 2003), giving an approximate seed concentration of 20 nM in a yeast cell, would result in a half-time of approximately 3 min. We observe Sup35 to be relatively stable against proteolysis within cells, so it would need to be replenished with a half-time of 90 min dictated by the doubling time of yeast. Excluded volume effects from the high concentration of proteins in cytoplasm may further increase the polymerization rate, as we found that 25% ficoll 70 increases polymerization rates by a factor of two or three. Also, 2 × 10^5^ M^−1^ s^−1^ may be an underestimate of the second-order rate constant if we underestimated the number of monomers per unit length in a fiber. We also note that fiber growth in the cell is not likely to be limited by conformational rearrangement after binding of monomer to fiber end because the concentration of Sup35 in a cell is in the neighborhood of 1 μM (Sparrer et al. 2000).

## Supporting Information

Protocol S1 Overview of Approach and Techniques Used (590 KB DOC).

### Accession Numbers

The GenBank accession number for Sup35p is NP_010457.

## Abbreviations

AFM: atomic force microscopy
AUC: analytical ultracentrifugation
GuHCl: guanidine hydrochloride
NM: a fragment of the Sup35 protein containing the glutamine/asparagine-rich N-terminal (N) and highly charged middle (M) domains
TIRF: total internal reflection fluorescence microscopy
