# Supplementary material for: Mechanism of Prion Propagation: Amyloid Growth Occurs by Monomer Addition
Source: PLoS Biol. 2004 Sep 21;2(10):e321. doi: 10.1371/journal.pbio.0020321 (PMC517824; doi:10.1371/journal.pbio.0020321)
Supplement: Protocol S1 — (590 KB DOC). [file pbio.0020321.sd001.doc]

# Overview of Approach and Techniques Used

## Importance of measuring direct parameters

A common practice used in studies including some of our previous work was to characterize polymerization reactions by parameters such as length of the lag time, length of time to go from 5% completion of polymerization to 95% completion (conversion time), and the maximal rate of the reaction (V_max_). From these measurements, attempts were made to infer details about the underlying kinetic processes. However, phenomenological parameters such as these, while easy to measure, can vary in highly complex ways depending on the underlying processes.


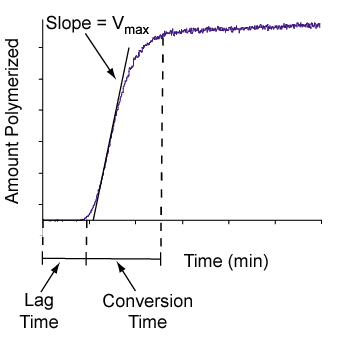


For example, the V_max_ of a reaction reflects both the rate of growth of fibers and the concentration of fibers present in the reaction at the time it hits its maximal rate. Doubling the growth rate of fibers may result in less than a doubling of the V_max_ of the reaction, as the reaction may now hit its maximum velocity with a smaller concentration of fiber ends. Thus, without more complete analysis or knowledge of the underlying mechanism, it is impossible to distinguish changes in growth rate of fibers from changes in rates of formation of new fibers. The lag time and conversion time of a reaction can similarly have complex nonlinear dependences on concentration, depending on the mechanism of the reaction.

For this reason, we tried to design measurements of individual mechanistic steps. From these measurements we then built a detailed mechanistic model and used this microscopic model to calculate properties of phenomenological parameters.

## Examining the growth step

Fiber growth (see diagram below) is an easily isolatable step in some systems, such as ours, because in the presence of a large quantity of fiber ends, growth occurs on a timescale much faster than formation of new fibers.


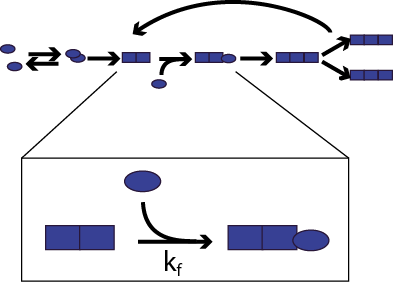


By measuring the initial rate of a seeded polymerization reaction (as shown below), one can readily determine a rate proportional to the growth rate of fibers.


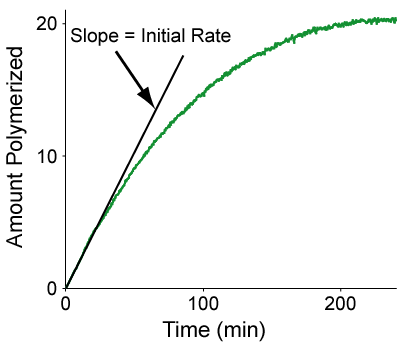


Then, by measuring concentration dependences of this initial rate, one can get direct mechanistic information about the growth step. For example, if this rate is proportional to seed concentration, then no step prior to interaction of soluble protein with fiber end is rate limiting. Additionally, the dependence of this rate on soluble concentration can give an indication of the size of the species adding to fiber ends. If a step after the binding of soluble protein to fiber end is limiting, this rate will show a less than first order dependence on soluble protein concentration. However, such a less than first order dependence could also be due to off-pathway aggregation. In this case the concentration of soluble, addition-competent protein would depend on less than the first power of the total soluble concentration.

## Examining curve shape

The shape of the polymerization curve can give significant insight into the underlying processes. For example, is the polymerization time course initially parabolic (t^2^) or exponential (e^t^) or does it show a different time dependence? An exponential time course is indicative of a secondary nucleation process such as fiber branching or fragmentation, whereas a t^2^ dependence is expected for nucleated polymerization. If a t^2^ dependence is not found, then the standard use of the concentration dependence of the length of the lag time to estimate nucleus size (nucleus size = 2n where lag time scales as the n^th^ power of concentration) should not be used.
